# Dysfunctional host cellular immune responses are associated with mortality in melioidosis

**DOI:** 10.1080/22221751.2024.2380822

**Published:** 2024-07-15

**Authors:** Shelton W. Wright, Peeraya Ekchariyawat, Sineenart Sengyee, Rungnapa Phunpang, Adul Dulsuk, Natnaree Saiprom, Ekkachai Thiansukhon, Kovit Pattanapanyasat, Sunee Korbsrisate, T. Eoin West, Narisara Chantratita

**Affiliations:** a Division of Pediatric Critical Care Medicine, Department of Pediatrics, University of Washington, Seattle, WA, USA; b Department of Microbiology, Faculty of Public Health, Mahidol University, Bangkok, Thailand; c Department of Microbiology and Immunology, Reno School of Medicine, University of Nevada, Reno, NV, USA; d Department of Microbiology and Immunology, Faculty of Tropical Medicine, Mahidol University, Bangkok, Thailand; e Department of Medicine, Udon Thani Hospital, Udon Thani, Thailand; f Department of Immunology, Faculty of Medicine Siriraj Hospital, Mahidol University, Bangkok, Thailand; g Center of Excellence for Microparticle and Exosome in Diseases, Department of Research and Development, Faculty of Medicine Siriraj Hospital, Mahidol University, Bangkok, Thailand; h Division of Pulmonary, Critical Care and Sleep Medicine, Department of Medicine, University of Washington, Seattle, WA, USA; i Department of Global Health, University of Washington, Seattle, WA, USA; j Mahidol-Oxford Tropical Medicine Research Unit, Faculty of Tropical Medicine, Mahidol University, Bangkok, Thailand

**Keywords:** Melioidosis, host immune response, cellular immunity, sepsis, LMIC

## Abstract

Melioidosis is a tropical infection caused by the intracellular pathogen *Burkholderia pseudomallei*, an underreported and emerging global threat. As melioidosis-associated mortality is frequently high despite antibiotics, novel management strategies are critically needed. Therefore, we sought to determine whether functional changes in the host innate and adaptive immune responses are induced during acute melioidosis and are associated with outcome. Using a unique whole blood stimulation assay developed for use in resource-limited settings, we examined induced cellular functional and phenotypic changes in a cohort of patients with bacteremic melioidosis prospectively enrolled within 24 h of positive blood culture and followed for 28 days. Compared to healthy controls, melioidosis survivors generated an IL-17 response mediated by Th17 cells and terminally-differentiated effector memory CD8^+^ T cells (*P* < .05, both), persisting to 28 days after enrolment. Furthermore, melioidosis survivors developed polyfunctional cytokine production in CD8^+^ T cells (*P* < .01). Conversely, a reduction in CCR6^+^ CD4^+^ T cells was associated with higher mortality, even after adjustments for severity of illness (*P* = 0.004). Acute melioidosis was also associated with a profound acute impairment in monocyte function as stimulated cytokine responses were reduced in classical, intermediate and non-classical monocytes. Impaired monocyte cytokine function improved by 28-days after enrolment. These data suggest that IL-17 mediated cellular responses may be contributors to host defense during acute melioidosis, and that innate immune function may be impaired. These insights could provide novel targets for the development of therapies and vaccine targets in this frequently lethal disease.

## Introduction

Melioidosis is a severe infection caused by the Gram-negative bacteria *Burkholderia pseudomallei* [1]. This tropical infection is classically associated with areas of Southeast Asia and northern Australia, where *B. pseudomallei* is known to be endemic [2]. However, melioidosis is likely underreported globally and recent autochthonous cases, including in inland China and North America, suggest it is a rapidly emerging disease [3–5]. Hospitalized patients often present with respiratory symptoms or evidence of shock, and death is common in northeastern Thailand, despite appropriate antibiotic treatment [1]. Given the severity of infection, *B. pseudomallei* is categorized by the United States Centers for Disease Control and Prevention as a Tier 1 bioterrorism agent. Diabetes and advanced age are major risk factors for melioidosis acquisition, and vaccine development efforts are ongoing [6].

In part to guide the development of novel therapeutics and vaccine targets, many investigative efforts have focused on the role of the host immune response during *B. pseudomallei* infection. A facultative intracellular pathogen, *B. pseudomallei* can survive and replicate in phagocytes and employs multiple different mechanisms to evade host clearance [7]. However, *B. pseudomallei* infection can also initiate a profound innate immune response, likely propagated initially by monocyte stimulation and subsequent local neutrophil recruitment [8,9]. Although bacterial clearance appears to be neutrophil-dependent, neutrophil activity may contribute to excessive tissue damage and inflammation [10–12]. The specific mechanisms underlying the adaptive immune response during *B. pseudomallei* infection have been a target of numerous in vitro models of melioidosis, in part to guide vaccine development [13,14]. As diabetes is a key risk factor for presentation with melioidosis and diabetics are likely a prime target group for vaccination, diabetes-associated dysregulation of T cell immune responses to melioidosis, particularly related to impaired IFN-γ production, has been well characterized [15–17].

In northeastern Thailand, mortality rates in patients with melioidosis can exceed 40%, despite appropriate antibiotic therapies [18]. Therefore, an urgent need exists to develop novel therapeutic approaches to this disease. Despite extensive in vitro and in vivo efforts to elucidate the innate and humoral responses complicit in *B. pseudomallei* infection, little is known about broader characteristics of the host immune response in patients with melioidosis. As the innate immune system may influence adaptive immunity, we therefore sought to determine associations between patient outcomes and immune cell phenotype and function in patients with melioidosis. Many prior reports regarding cellular responses in patients with melioidosis have utilized cryopreserved peripheral blood mononuclear cells (PBMC), and functional responses have typically focused on T cells using an enzyme-linked immunospot (ELISpot) assay targeting IFN-γ [13,15–17]. However, cryopreservation and isolation of PBMCs may alter functionality, including after stimulation, limiting assessments of the innate immune system [19]. Additionally, prior cellular response efforts have not typically included evaluations of antigen-presenting cell functionality or cellular polyfunctionality. In this study, we employed a premade whole blood assay, designed to stimulate cells prior to cryopreservation, for utilization in a resource-limited setting, allowing for downstream single-cell phenotyping and functional assessments [20]. The goal of this study was to determine whether innate and adaptive immune cell phenotypes and function change over time in patients with melioidosis and are associated with mortality. Therefore, we performed single-cell analyses on peripheral blood from prospectively enrolled patients with melioidosis at the time of enrolment and 28 days later and compared cellular responses to healthy volunteers.

## Methods

### Study design and participants

Study enrolment occurred at Udon Thani Hospital in Udon Thani, Thailand. Hospitalized patients at least 15 years of age were prospectively enrolled within 24 h of the identification of a positive blood culture for *B. pseudomallei* between December 2016 and July 2018. Patients were identified by daily screening in the hospital microbiology laboratory. This sub-cohort is part of a larger, previously described cohort [6]. Clinical data was obtained from the medical record and study participants or their surrogates. At the time of enrolment, whole blood was obtained. Additionally, blood was obtained 28 days after enrolment from those surviving to that timepoint. A healthy control cohort of volunteer adults ≥18 years of age without any chronic medical conditions, history of melioidosis or recent vaccination or infectious symptoms was recruited from the Udon Thani Hospital blood donation centre.

### Preparation of blood stimulation plates

Premade 96-well plates, intended for cellular stimulation prior to cryopreservation and containing 20 µl of *B. pseudomallei* LPS (Bp-LPS, 10 ng/ml) or RPMI media alone, were prepared at Faculty of Tropical Medicine, Mahidol University, Bangkok, Thailand, using validated protocols [20–23]. Bp-LPS was prepared as previously described [24]. All wells contained brefeldin A (Sigma-Aldrich, St. Louis, MO, USA) at 10 µl/ml. The plates were sealed, stored at −80°C and shipped to the study site on dry ice monthly, where they were stored at −20°C for a maximum of one month prior to use.

### Whole blood processing

At the time of procurement from study participants, whole blood was mixed 1:1 with pre-warmed RPMI 1640 media. Following mixing, 180 µl of diluted whole blood was placed into the premade 96-well plates, some of which contained Bp-LPS for stimulation. Plates were immediately incubated at 37°C for 6 h. Viability dye, eFluor 780 (eBioscience), was then added to each sample and incubated for 30 min at 4°C. Following incubation, erythrocytes were lysed with 200 µl of Stable-Lyse V2 solution (Smart Tube, Las Vegas, NV, USA) and incubated 15 min at room temperature. Following incubation, 500 µl of Stable-Store V2 solution was added and samples were cryopreserved.

Thawed samples were centrifuged at 600 g for 5 min and then resuspended in cold FACS buffer (PBS with 0.5% bovine serum albumin and 0.1% sodium azide). Samples were incubated in 100 µl Perm II buffer (BD Biosciences, San Jose, CA) for 30 min, centrifuged and resuspended in FACS buffer. Cells were stained with a mixture of fluorophore-conjugated antibodies (Supplementary Table 1) for 30 min and then resuspended in FACS buffer. Antibody panels were optimized prior to use and cell populations identified using a fluorescence minus one (FMO) control; intracellular cytokine antibodies were optimized using stimulation with phorbol 12-myristate 13-acetate (PMA) and ionomycin. Data were acquired on a BD LSR II flow cytometer at Mahidol University, Bangkok, Thailand and analysed using FlowJo software (BD). The gating strategies can be seen in Supplementary Figures 1 and 2.

Cellular populations were identified using cell surface markers after live cell gating. For lymphocytes, these included CD4^+^ T cells (CD3^+^, CD4^+^), CD8^+^ T cells (CD3^+^, CD8^+^), and Th17 cells (CD4^+^, CCR6^+^, IL-17^+^). Lymphocyte phenotype was determined by cell surface expression of CD45RA and CCR7: effector memory (TEM: CD45RA^-^, CCR7^-^) and terminally differentiated effector memory T cells (TEMRA: CD45RA^+^, CCR7^-^). Non-lymphocytes identified included granulocytes (HLA-DR^-^, CD14^+^), NK cells (CD3^-^, CD16/CD56), conventional dendritic cells (HLA-DR^+^, CD14^-^, CD11c^+^, CD123^-^), plasmacytoid dendritic cells (HLA-DR^+^, CD14^-^, CD11c^-^, CD123^+^) and B cells (HLA-DR^+^, CD14^-^, CD11c^-^, CD123^-^). Finally, populations of monocytes were identified including classical monocytes (HLA-DR^+^, CD14^+^, CD16^-^), non-classical monocytes (HLA-DR^+^, CD14^-^, CD16^+^), and intermediate monocytes (HLA-DR^+^, CD14^+^, CD16^+^).

### Statistical analysis

Normally distributed data are reported as mean and standard deviation and non-normally distributed data are reported as median and interquartile range (IQR). Comparisons of normally distributed data were made using Student's *t*-test or ANOVA with Tukey's test for multiple comparisons. Non-normally distributed data comparisons were made using the Mann–Whitney *U* test or Kruskal–Wallis test with Dunn's test for multiple comparisons. Paired samples were compared using either a paired *t*-test for normally distributed data or a Wilcoxon test for non-normally distributed data. T cell polyfunctional data was acquired using Boolean combination gating and indices calculated using published models [25]. Logistic regression models were developed including those adjusting for (1) baseline risk factors (age, reported sex, history of diabetes and history of chronic kidney disease), (2) baseline risk factors and enrolment lymphocyte count (based on hospital complete blood count differential) and finally (3) baseline risk factors, enrolment lymphocyte count and evidence of critical illness, including enrolment requirement of mechanical ventilation or vasoactive support. Statistical analyses were performed using Prism software (GraphPad, La Jolla, CA) and Stata SE version 14.2 (StataCorp, College Station, TX). *P* < .05 was considered statistically significant.

### Ethics approval

The study was approved by the ethics committees of Faculty of Tropical Medicine, Mahidol University (MUTM2015-002-01) and Udon Thani Hospital (UD 0032.102/318). The University of Washington issued a statement of non-engagement. The study was conducted according to the principles of the Declaration of Helsinki (2008) and the International Conference on Harmonization (ICH) Good Clinical Practice (GCP) guidelines. Written informed consent was obtained from all patients enrolled in the study.

## Results

### Patient characteristics

Thirty-two prospectively enrolled patients with melioidosis were included in the analysis. In this patient set, the median age was 55 years (IQR 39-64) and 41% (13/32) identified as female (Table 1). Diabetes was common as 59% (19/32) had been diagnosed prior to presentation. The patient set had a high severity of illness at enrolment with 44% (14/32) requiring mechanical ventilation and 47% (15/32) requiring vasoactive support. In this severely ill patient set, 31% (10/32) died within 28 days of enrolment. In the healthy control cohort (*N* = 10), the median age was 49 years (IQR 41-56) and 50% identified as female.

**Table 1.** Melioidosis patient characteristics at enrolment.

Characteristics	Set (*N* = 32)
*Demographics*	
Age in years, median (IQR)	55 (39-64)
Self reported as female, *N* (%)	13 (41)
*Pre-existing conditions*	
Diabetes, *N* (%)	19 (59)
Chronic liver disease, *N* (%)	0 (0)
Chronic kidney disease, *N* (%)	6 (19)
Chronic cardiovascular disease, *N* (%)	1 (3)
Chronic lung disease, *N* (%)	1 (3)
Cancer, *N* (%)	1 (3)
HIV, *N* (%)	0 (0)
Chronic steroids, *N* (%)	4 (13)
Days from admission to enrolment, median (IQR)	4.5 (3-6)
Severity of illness	
Mechanical ventilation, *N* (%)	14 (44)
Vasoactive medication requirement, *N* (%)	15 (47)
Died by 28-days, *N* (%)	10 (31)

### Relative decline in CD4^+^ T cell populations is associated with death in melioidosis

**Figure 1.**
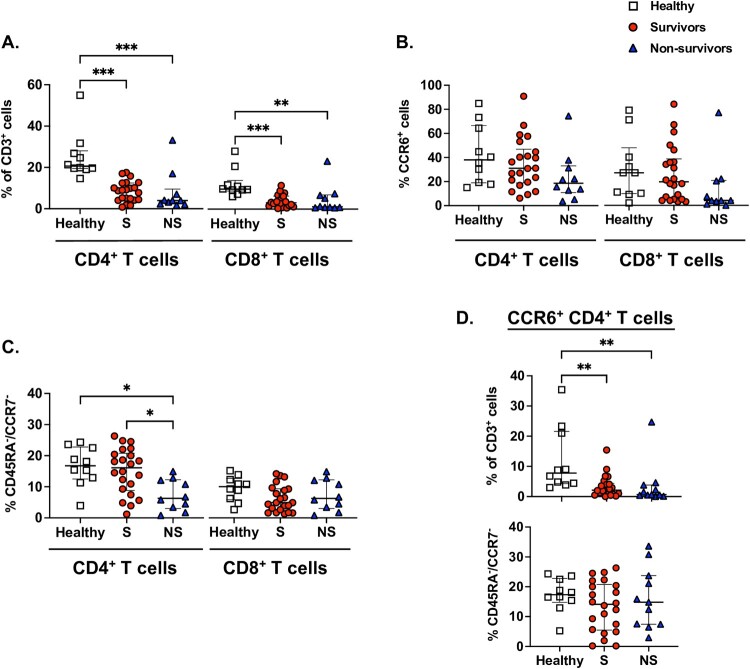
T cell phenotypes shift in acute melioidosis. Whole blood was obtained from patients with melioidosis within 24 h of culture positivity (Survivors: S, *N* = 22; 28-day non-survivors: NS, *N* = 10) and healthy donors (Healthy, *N* = 10). (A) CD4^+^ and CD8^+^ cells were quantified as percentages of live CD3^+^ cells in unstimulated blood using flow cytometry. T cell phenotypes were determined by measuring the percentage of CD4^+^ and CD8^+^ T cells which were (B) CCR6^+^ and (C) CD45RA^-^, CCR7^-^. (D) CD4^+^, CCR6^+^ T cells are shown as a percentage of live CD3^+^ cells and the CD45RA^-^, CCR7^-^ effector memory phenotype was quantified as a percentage of CCR6^+^ CD4^+^ cells . Median and interquartile range presented; the Kruskal–Wallis test was performed for statistical comparisons, followed by the Dunn's test for multiple comparisons. *P < .05, **P < .01, ***P < .001.

A recent study using cryopreserved PBMCs suggested that melioidosis mortality is associated with a lower CD8^+^ T cell concentration [15]. As our premade whole blood stimulation technique may allow for a unique evaluation of cellular functional phenotypes, we first assessed the proportion (Figure 1) and concentration (Supplementary Figure 3) of T-cell populations in patients with melioidosis and a healthy control set. Patients with melioidosis, regardless of outcome status, had significantly reduced proportions of both CD4^+^ and CD8^+^ T cells in blood compared to healthy controls (Figure 1(A)). A multitude of T cells express CCR6, including Th17 cells, and expression may impact inflammatory migration [26]. As cytotoxic cell migration may play an important role during an intracellular infection such as acute melioidosis, we next assessed the proportion of cells positive for CCR6 amongst T cell populations (Figure 1(B)). Though a trend towards reduced CCR6 expression was noted in both CD4^+^ and CD8^+^ T cells populations in melioidosis non-survivors, no significant difference was observed. As environmental exposure to *B. pseudomallei* is common in endemic regions, we postulated that the mobilization of effector memory (TEM: CD45RA^-^ CCR7^-^) T cells could also be critical during acute melioidosis [27]. We found that melioidosis non-survivors had a significantly lower proportion of CD4^+^ T cells with an effector, memory phenotype compared to melioidosis survivors (*P* < .05, Figure 1(C)). Additionally, this CD4^+^ T cell effector, memory phenotype was significantly reduced in melioidosis non-survivors compared to healthy controls. No difference in the proportion of TEMRA (terminally differentiated effector memory T cells: CD45RA^+^, CCR7^-^) cells was noted between patients with melioidosis and healthy controls.

As CCR6^+^ CD4^+^ T cells, including Th17 cells, may have specific inflammatory roles, we also assessed the proportion and concentration of CCR6^+^ CD4^+^ T cells in patients with melioidosis. Both melioidosis survivors and non-survivors had significantly reduced proportions of CCR6^+^ CD4^+^ T cells compared to healthy controls (both, *P* < .05; Figure 1(D)). Additionally, non-survivors had a significantly lower concentration of CCR6^+^ CD4^+^ T cells compared to survivors (Supplementary Figure 3C, *P* < .05).

We next determined the association of CD4^+^, CD8^+^ and CCR6^+^ CD4^+^ T cell concentrations with outcome at 28 days by developing logistic regression models. Peripheral blood lymphocyte counts in bacterial infections may be affected by multiple covariates including comorbidities and severity of illness. Therefore, we developed models with multiple adjustments, including baseline risk factors (age, history of diabetes, and history of chronic kidney disease), enrolment lymphocyte count and need for either mechanical ventilation or vasoactive medications at enrolment. CD4^+^ and particularly CCR6^+^ CD4^+^ T cell concentrations were inversely associated with the odds of death at 28 days in models progressively adjusting for baseline risk factors, enrolment lymphocyte count and critical illness at the time of enrolment (CD4^+^: adjusted odds ratio (AOR) 0.98, 95% confidence interval (CI) 0.96-1.00, *P* = .02; CCR6^+^ CD4^+^: AOR 0.92, 95% CI 0.87–0.98, *P* = .004; Table 2). CD8^+^ T cell concentration was not associated with mortality. The association of a lower CCR6^+^ CD4^+^ T cell concentration with higher mortality, even after adjustment, suggests the involvement of these cells in the host immune response to melioidosis.

**Table 2.** Association of blood T cell concentration and 28-day mortality.

	Adjusted for baseline risk factors^a^			Adjusted for baseline risk factors and lymphocyte count^b^			Adjusted for baseline risk factors, lymphocyte count and critical illness^c^		
Cells/μl	AOR	95% CI	*P*	AOR	95% CI	*P*	AOR	95% CI	*P*
CD4^+^	0.98	0.96–0.99	0.01	0.98	0.96–0.99	0.01	0.98	0.96–1.00	0.02
CD8^+^	0.98	0.95–1.02	0.28	0.98	0.95–1.01	0.20	0.98	0.96–1.00	0.10
CCR6^+^, CD4^+^	0.93	0.88–0.97	0.003	0.93	0.88–0.98	0.009	0.92	0.87–0.98	0.004

Abbreviations: AOR: adjusted odds ratio; CI: confidence interval; *P*: *P*-value.

^a^ Baseline logistic regression models adjusted for age, sex, history of diabetes, and history of chronic kidney disease.

^b^ Logistic regression models adjusted for lymphocyte concentration from clinical laboratory white blood cell differential count of lymphocytes obtained at the time of enrolment and baseline risk factors.

^c^ Logistic regression models adjusted for vasopressor usage and/or mechanical ventilation, lymphocyte count at the time of enrolment, and baseline risk factors.

**Figure 2.**
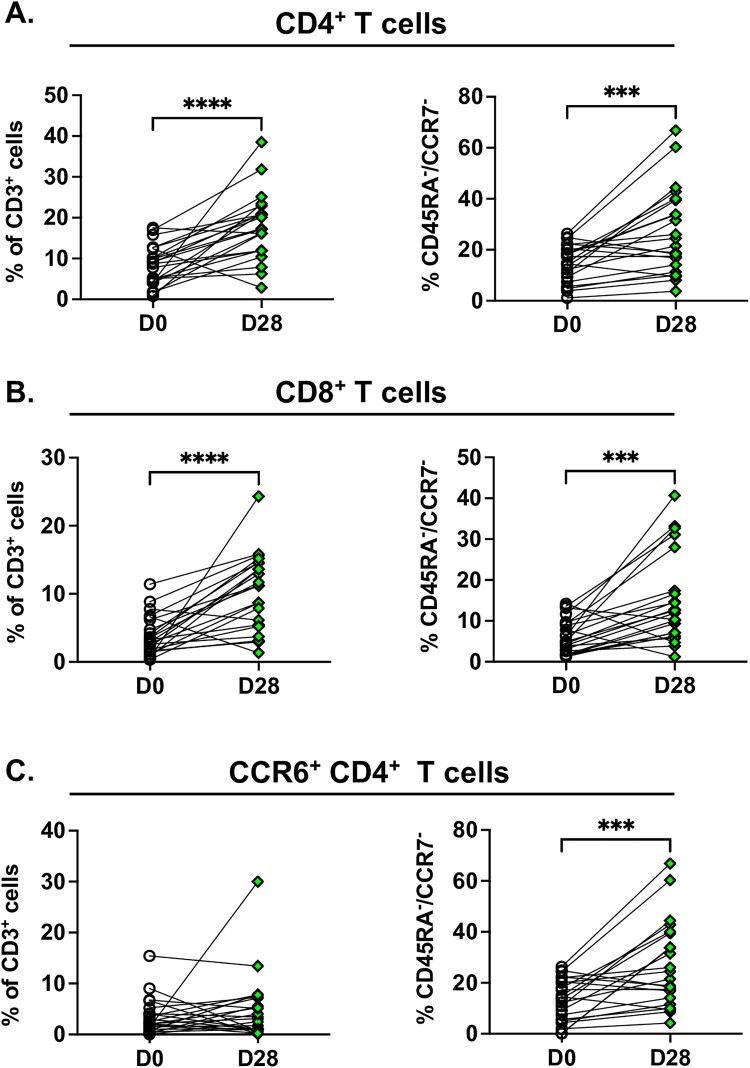
T-cell phenotypes demonstrate recovery after melioidosis survival. Whole blood was obtained from patients with melioidosis at the time of study enrolment within 24 h of culture positivity (D0) and at 28 days after enrolment (D28, *N* = 22). Flow cytometry was performed on unstimulated whole blood to determine changes over time in (A) CD4^+^, (B) CD8^+^ and (C) CCR6^+^ CD4^+^ T cells as percentages of live, CD3^+^ cells and the percentage of each cell type with a CD45RA^-^, CCR7^-^ phenotype. Median and interquartile range presented; the Wilcoxon test for paired samples was used for statistical analysis. ***P < .001, ****P < .0001.

Finally, as we had identified several cell populations associated with outcome, we evaluated whether T cell subsets and phenotypes change over the course of infection. In patients surviving to 28 days after enrolment, the proportion of CD4^+^ and CD8^+^ T cells had recovered by day 28 (both, *P* < .001; Figure 2(A,B)). Additionally, both CD4^+^ and CD8^+^ T cells had a propensity for developing a CD45RA^-^ CCR7^-^ effector memory phenotype by day 28 (both, *P* < .001), suggesting a possible role in immunity against re-infection in the event of future exposure to *B. pseudomallei*. While the proportion of CCR6^+^ CD4^+^ T cells did not change over 28 days, they were also more likely to develop an effector memory phenotype over this timeframe (*P* < .001; Figure 2(C)).

### Melioidosis survival is associated with enhanced effector T cell function after ex vivo stimulation

**Figure 3.**
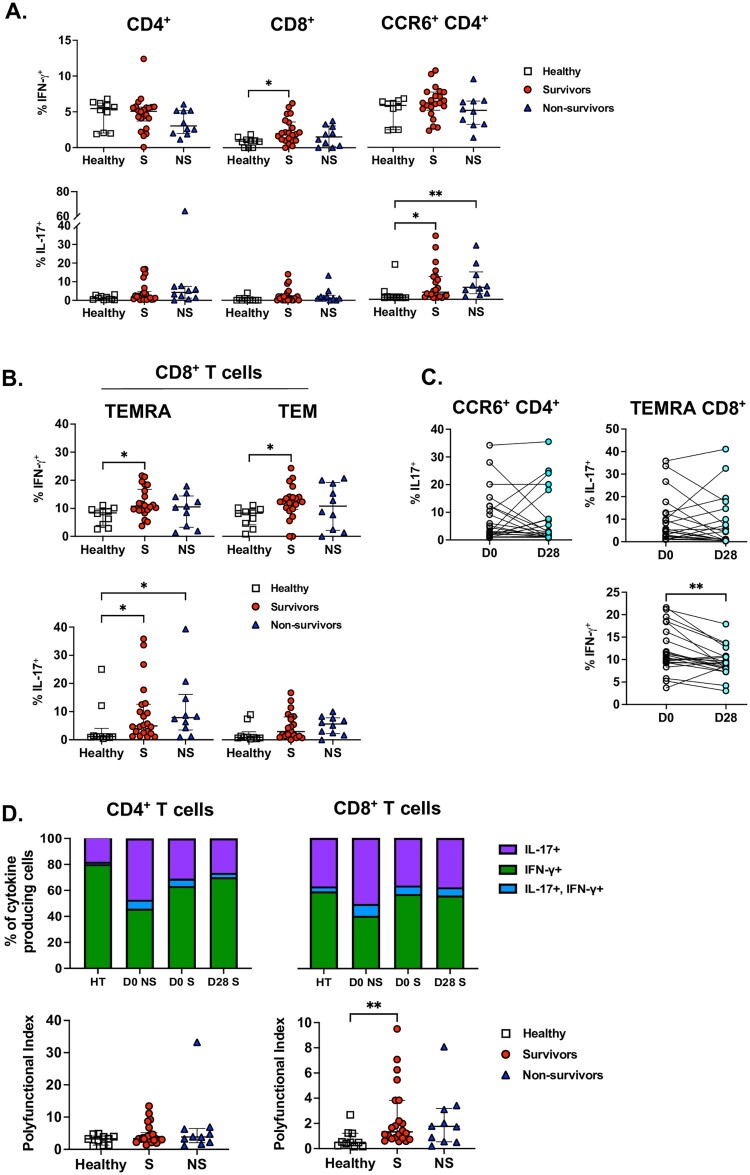
Melioidosis survivors have increased T cell cytokine activity. Whole blood was obtained from patients with melioidosis within 24 h of culture positivity (Survivors: S/D0 S, *N* = 22; 28-day non-survivors: NS/D0 NS, *N* = 10) and healthy donors (Healthy/HT, *N* = 10). Whole blood samples were immediately stimulated with Bp-LPS, stained for surface and intracellular markers and assessed by flow cytometry. (A) T-cell populations were quantified by the percentage of each cell population positive for IFN-γ and IL-17. (B) The distribution of cells producing IFN-γ or IL-17 was determined in terminally differentiated effector memory (TEMRA, CD45RA^+^, CCR7^-^) and effector memory (TEM, CD45RA^-^, CCR7^-^) CD8^+^ T cells. (C) Whole blood obtained at 28-days after enrolment (D28) was compared to enrolment (D0) samples for the proportion of cytokine-producing CCR6^+^ CD4^+^ T cells and TEMRA CD8^+^ T cells. (D) Amongst CD4^+^ and CD8^+^ T cells that produced cytokines, graphs display the mean proportion of cells producing IL-17 alone, IFN-γ alone or both. Additionally, a polyfunctional index was calculated to quantify stimulated CD4^+^ and CD8^+^ T cell cytokine production. Except as noted above, median and interquartile range presented; the Kruskal–Wallis test was performed for statistical comparisons, followed by the Dunn's test for multiple comparisons. *P < .05, **P < .01.

Peripheral blood mononuclear cells from melioidosis non-survivors are reported to release less IFN-γ after stimulation with inactivated *B. pseudomallei* compared to survivors; though the overall proportion of CD4^+^ or CD8^+^ T cells that produce IFN-γ is low [16]. As our study design might preserve cellular function differently compared to prior studies, we next examined the responses of T cells after stimulation with Bp-LPS. The Bp-LPS stimulus was chosen over other stimuli as it had better suitability in the premade plates than heat-killed *Burkholderia pseudomallei*, because it is an established cellular agonist that we have studied extensively, and because unlike phorbol 12-myristate 13-acetate (PMA) and ionomycin, it is specific to *Bps* infection [24,28]. Additionally, IL-17, a cytokine frequently produced by certain lymphocyte populations, may play a role in vaccine responses for *B. pseudomallei* but data regarding its cellular sources in clinical melioidosis is limited [29]. Therefore, to assess adjuvant T cell activation and functional differentiation, we assessed intracellular IFN-γ and IL-17 in different T cell populations [30]. CD8^+^, but not CD4^+^, T cells in melioidosis survivors were significantly more likely to produce intracellular IFN-γ in response to Bp-LPS stimulation than healthy controls (*P* < .05; Figure 3(A)). No difference was noted in the proportion of CD4^+^ or CD8^+^ T cells that produced IL-17 after Bp-LPS stimulation. However, the frequency of CCR6^+^ CD4^+^ T cells staining for IL-17-denoting these cells as likely Th17 cells-was significantly higher in patients with melioidosis compared to healthy controls (*P* < .05; Figure 3(A)). In order to further characterize cytokine-producing T cells, we next measured cytokine production by both effector memory (TEM; CD45RA^-^, CCR7^-^) and terminally differentiated (TEMRA; CD45RA^+^, CCR7^-^) effector memory T cells. The proportion of TEMRA CD8^+^ T cells producing either IFN-γ or IL-17 was significantly higher in melioidosis survivors compared to healthy controls (Figure 3(B), *P* < .05). No difference in cytokine production was noted in CD4^+^ effector memory T cells. Intriguingly, while the proportion of IL-17-producing CCR6^+^ CD4^+^ T cells or TEMRA CD8^+^ T cells did not change by 28 days after infection, the proportion of IFN-γ-producing TEMRA CD8^+^ T cells declined by 28 days (*P* < .01, Figure 3(C)).

As T-cell polyfunctionality may identify distinct populations with critical roles during infection, we next evaluated T cell cytokine production frequency [31]. In cytokine-producing CD4^+^ and CD8^+^ T cells, polyfunctionality was not common and, both CD4^+^ and CD8^+^ cells from non-survivors tended to be more likely to produce IL-17 compared to healthy controls or survivors (Figure 3(D)). Additionally, compared to healthy controls, the CD8^+^ T cells of survivors had a significantly higher polyfunctional index (*P* < .01, Figure 3(D)), a method of quantifying the extent and variation of polyfunctionality, allowing for comparative analysis [20,25]. Taken together, these data suggest that Th17 and IL-17-producing TEMRA CD8^+^ T cells are upregulated in acute melioidosis and persist after infection. Additionally, CD8^+^ T cells from melioidosis survivors are most capable of IFN-γ and IL-17 polyfunctionality.

### Host innate immune cell populations are differentially regulated in acute melioidosis

**Figure 4.**
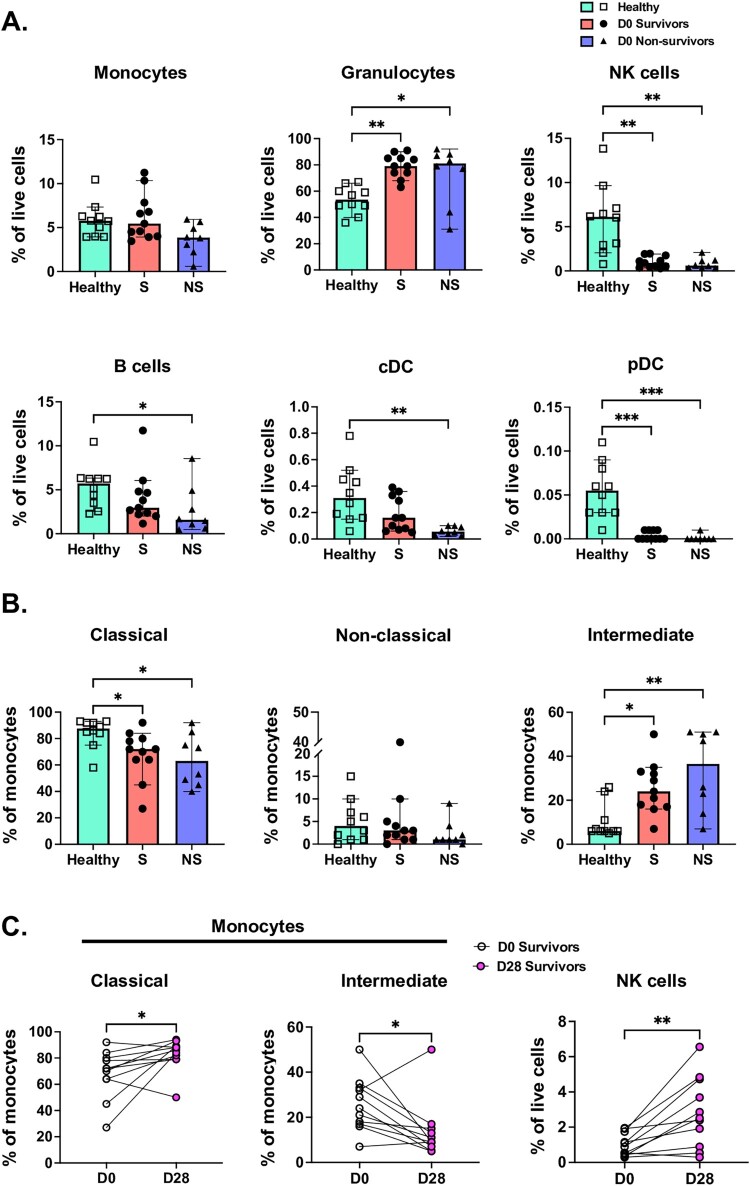
Whole blood was obtained from patients with melioidosis within 24 hours of culture positivity (Survivors: S (circles), *N* = 11; 28-day non-survivors: NS (triangles), *N* = 8) and healthy donors (Healthy (squares), *N* = 10). (A) Unstimulated whole blood was assessed by flow cytometry for different immune cell populations as a percentage of live cells. (B) Monocyte populations including classical (CD14^+^, CD16^-^), non-classical (CD14^-^, CD16^-^) and intermediate (CD14^+^, CD16^+^) monocyte populations were also identified. (C) Whole blood was obtained from patients with melioidosis at 28-days after enrollment (D28/solid circles) and compared to whole blood obtained from the same patients at the time of enrollment (D0/open circles, *N* = 11) for the relative frequency of innate cell populations. Median and interquartile range presented; the Kruskal-Wallis test was performed for statistical comparisons of unpaired data, followed by the Dunn's test for multiple comparisons. The Wilcoxon test was used for comparisons of paired samples. *P < 0.05, **P < 0.01, ***P < 0.001.

The host innate immune response appears critical in melioidosis, and antigen-presenting cells (APC) are likely crucial for the activation of T-cell responses. Therefore, we next measured the relative frequencies (Figure 4) and concentrations (Supplementary Figure 4) of common APCs for *B. pseudomallei*, including monocytes and dendritic cells, in a subset of the primary patient set (*N* = 19, of whom 8 died before 28-days). Additionally, as granulocytes, NK cells, and B cells may influence APCs within the host immune response during bacterial infections, these populations were quantified as well. Not surprisingly, melioidosis was associated with a significantly higher proportion (and concentration) of granulocytes, though no survival difference was noted (Figure 4A, Supplementary Figure 4A). Conversely, relative frequencies of B cells, cDC and pDC were significantly lower in melioidosis non-survivors compared to healthy controls, though these represented a small fraction of live cells (*P* < .05, all). NK cell frequencies were significantly lower in both survivors and non-survivors compared to healthy controls (*P* < .01). The relative reduction in these cell populations in melioidosis patients relative to healthy controls may be related to neutrophil recruitment in the setting of an acute bacterial infection. Additionally, we also sought to characterize the relative proportions of monocyte subsets. Although the overall monocyte percentage was similar in melioidosis patients and healthy controls, the relative proportion of classical monocytes (CD14^+^, CD16^-^) was lower in patients with melioidosis (*P* < 0.05, Figure 4B). Notably, while intermediate monocytes (CD14^+^, CD16^+^) make up a small proportion of circulating monocytes in healthy controls, the proportion of these cells was significantly higher in patients with melioidosis (*P* < .05). These data suggest that melioidosis may induce a relative upregulation of intermediate monocytes at the expense of classical monocytes. We postulated that relative cell populations would likely recover to baseline trends 28 days after enrolment in survivors, particularly as circulating neutrophil counts would be anticipated to decline over this time. By 28 days after enrolment, the relative frequency of granulocytes (*P* < .01) decreased and NK cells (*P* < .01; Figure 3C), cDC and pDC populations also proportionally increased in survivors. B cell and monocyte frequencies remained stable over time in survivors. Additionally, the proportion of classical and intermediate monocytes also reverted to near healthy baselines (*P* < .05, both).

### Monocyte cytokine responses are suppressed in melioidosis

**Figure 5.**
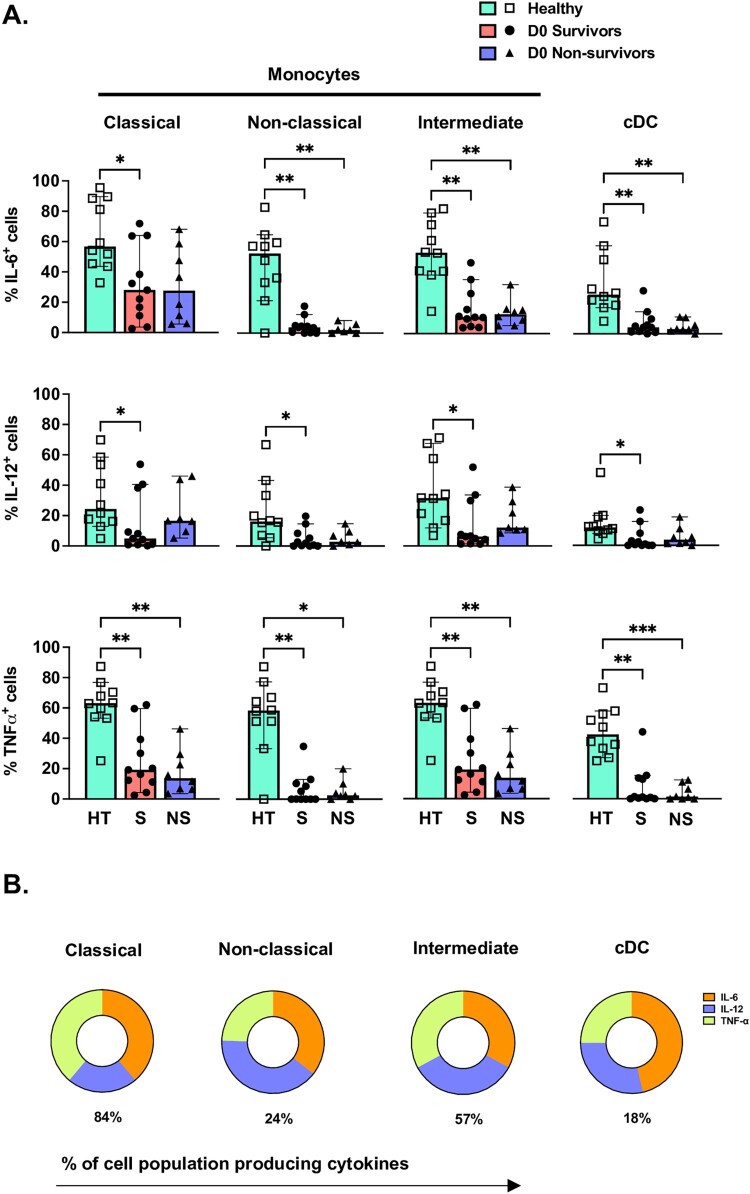
Whole blood was obtained from patients with melioidosis within 24 hours of culture positivity (Survivors: S (circles), *N* = 11; 28-day non-survivors: NS (triangles), *N* = 7–8) and healthy donors (Healthy (squares), *N* = 10). Whole blood samples were immediately stimulated with Bp-LPS, stained for surface and intracellular markers and assessed by flow cytometry. (A) Monocyte and cDC populations were quantified by the percentage of each cell population positive for IL-6, IL-12 and TNF-α. (B) Amongst monocyte and cDC populations that were cytokine producing, circle graphs display mean proportion of cells producing IL-6, IL-12 and TNF-α. Except as noted above, median and interquartile range presented; the Kruskal-Wallis test was performed for statistical comparisons, followed by the Dunn's test for multiple comparisons. *P < 0.05, **P < 0.01, ***P < 0.001.

As the proportional changes in innate immune cell frequency in melioidosis could be related to the profound circulating neutrophil response during infection, we next sought to assess whether innate cell cytokine responses may be affected during acute melioidosis. As monocyte subset populations were significantly altered in melioidosis, we assessed their relative intracellular cytokine production after stimulation by Bp-LPS. Relative intracellular production of IL-6, IL-12, TNF-α and IFN-γ were measured as these cytokines can be expressed by monocytes and might affect T cell responses. Additionally, as cDC's may also play an important APC role in melioidosis and could produce this cytokine array, these cells were also assessed. In general, monocytes responded to stimulation with Bp-LPS by most frequently producing IL-6 and TNF-α, followed by IL-12 (Figure 5A-B). Minimal IFN-γ production was observed in all populations. Nevertheless, the percentage of IL-6, IL-12 or TNF-α-producing monocytes was significantly lower in melioidosis patients relative to healthy controls. No difference in cytokine-producing cell frequency was observed between survivors and non-survivors. As decreased monocyte cytokine production after stimulation in cells obtained from acutely ill patients could be an indication of infection-induced immunosuppression or exhaustion, we postulated that cell function would improve over time, when surviving patients would be likely to be recovering. Indeed, in surviving patients, all monocyte subsets demonstrated a significant increase in their percentage of IL-6 and TNF-α-producing cells after 28 days (Supplementary Figure 5).

Finally, we explored the relationship between circulating monocyte and T cell concentrations in patients with melioidosis. Both CD4^+^ (Spearman's rho 0.53, *P* = .02) and CD8^+^ T cells (Spearman's rho 0.59, *P* = .01) were moderately correlated with non-classical monocyte concentrations (Supplementary Figure 6). Additionally, CD4^+^ T cells were moderately correlated with intermediate monocyte concentrations (Spearman's rho 0.60, *P* = .01).

## Discussion

In this study, we report that suppression of specific T cell populations circulating in blood is associated with worse outcomes in melioidosis. Additionally, we report that functional blood monocyte responses are suppressed during melioidosis and recover after illness. Our findings further implicate the T cell response during the acute phase of melioidosis but also highlight broader innate immune dysregulation, even in patients surviving the disease.

The importance of the T cell response during melioidosis has been an area of active investigation, particularly as a target of vaccines. Although lymphocyte populations rapidly decline during severe *B. pseudomallei* infection, as an intracellular pathogen it may be susceptible to T cell responses in addition to the innate immune response. For example, an animal model of melioidosis immunization suggested that CD4^+^ T cells mediated immunity specifically related to the Type III secretion system proteins *B. pseudomallei* uses during intracellular invasion [32]. Functional studies of T cells in melioidosis have typically focused on the production of IFN-γ, perhaps in part related to its ease of quantification and similarities between *B. pseudomallei* and *Mycobacterium tuberculosis*. In fact, depressed T cell function measured by IFN-γ production has previously been associated with melioidosis survival in several studies [16,17,33]. Furthermore, IFN-γ-related transcriptomic pathways are strongly upregulated in melioidosis, and IFN-γ appears to be necessary for survival in animal models of melioidosis [34,35]. In our study, we did not observe a difference in IFN-γ^+^ T cell responses between survivors and non-survivors. However, we did note that melioidosis survivors demonstrated an increased CD8^+^ IFN-γ response compared to healthy controls, but this upregulation was not observed in non-survivors. Additionally, the source of this CD8^+^ IFN-γ response appeared to be both TEMRA and TEM CD8^+^ cells. Previously, TEMRA CD8^+^ and CD4^+^ cells, but not TEM cells, were reported to be the primary IFN-γ producing lymphocytes after recovery from melioidosis [14]. Our results suggest the lymphocyte source of IFN-γ may be more heterogenous during active infection, though the generalization of this finding is limited by the usage of Bp-LPS rather than other stimuli and may represent indirect stimulation. Notably, impaired IFN-γ responses have been postulated as a mechanism explaining increased risk imparted by diabetes on melioidosis acquisition [15]. However, diabetes is also paradoxically associated with a reduced risk of death, including in a recent cohort study of over 1,300 patients with melioidosis [6]. As our interest in this study was related to immune correlates of survival, we did not specifically target diabetic patients to assess functional T-cell impairment.

In contrast to the robust literature regarding the role of IFN-γ, assessments of other T cell functional responses, including phenotypic characterization or alternative cytokine responses, are limited in prior reports on melioidosis. In our study, we report for the first time that the relative frequency of Th17 (CCR6^+^ CD4^+^ IL17^+^) cells is significantly increased in patients with melioidosis. Additionally, a lower concentration of peripheral blood CCR6^+^ CD4^+^ T cells was associated with an increased risk of death, even when adjusting for severity of illness and lymphocyte concentration. The role of IL-17-producing cells in melioidosis is unclear, but they appear important. For example, a sustained elevation in the concentration of circulating IL-17, of which Th17 cells are often a major source, is associated with worse outcomes in melioidosis [36]. Additionally, transcriptional pathways related to Th17 cell differentiation and IL-17 signalling are upregulated in melioidosis non-survivors compared to survivors [37]. Conversely, in two recent in vivo studies, a candidate glycoconjugate vaccine for *B. pseudomallei* induced a protective IL-17 response, and recombinant *B. pseudomallei*-associated proteins elicited an IL-17-associated T-cell response [29,38]. Suggestive of heterogenous sources of IL-17 during acute melioidosis, we found that the proportion of TEMRA CD8^+^ cells producing IL-17 significantly increased in both survivors and non-survivors. A possible role of IL-17 in melioidosis may relate to its role as a chemoattractant for neutrophils, and Th17 cells can directly influence neutrophil responses [39]. As the neutrophil response appears critical to bacterial clearance of *B. pseudomallei*, IL-17-producing cells in acute melioidosis may activate this process [40]. However, further work is needed to understand the specific role of IL-17-producing cells in acute melioidosis.

Although a developing area of study, the ability of T cells to simultaneously produce multiple cytokines has been associated with improved outcomes in several infectious diseases [41,42]. Additionally, T cells able to mount a polyfunctional phenotype may represent a distinct T cell population whose function is not pathogen-dependent [31]. In our study, we found that survivors of melioidosis were more likely to have an increased CD8^+^ polyfunctional index relative to healthy controls but non-survivors did not. While this may represent a unique inflammatory response to melioidosis, this lack of acquisition of a polyfunctional T cell response could also represent T cell exhaustion. Evidence of pathologic T cell exhaustion is limited in melioidosis but increased T cell expression of PD-1 can be induced during *B. pseudomallei* infection in vitro [43,44]. In our study, the overall increase in both T cell population frequency and effector memory phenotype over time in survivors suggests that any active T cell suppression during acute infection improves within weeks, and that T cell memory responses that develop may potentially provide immunity in the event of future exposure to *B. pseudomallei*.

Innate immune cell suppression is a relatively recently characterized phenomenon in sepsis [45,46]. Lower HLA-DR expression in monocytes, for example, correlates with reduced stimulated intracellular cytokine production and may identify patients at risk of developing sepsis-induced immune paralysis [47]. While similar evidence is limited in melioidosis, classical monocytes may have lower HLA-DR expression in melioidosis non-survivors compared to survivors [15]. Extending these findings in our study, we found that monocyte populations from melioidosis patients, particularly classical and intermediate monocytes, have reduced stimulated intracellular cytokine production. Additionally, we observed that these reduced monocyte responses recover within 28 days, suggesting acute infection-mediated suppression. Although reduced monocyte responses have been associated with worse outcomes in sepsis, we did not find a response difference between melioidosis survivors and non-survivors [48]. However, our data do suggest that monocyte production of IL-6 and TNF-α are profoundly affected during melioidosis. *B. pseudomallei* may inhibit NF-κB and type I IFN pathways in vitro potentially inhibiting monocyte cytokine production, postulated as a mechanism for host immune system evasion [49]. However, acute melioidosis is also associated with elevated circulating cytokine concentrations of IL-6 and TNF-α, and higher concentrations of these cytokines are associated with worse outcomes [36,50]. Additionally, human monocytes produce TNF-α in vitro in response to *B. pseudomallei* infection through Toll-like receptor 4, for which LPS is a primary ligand [9]. However, our results suggest that LPS-stimulated monocytes from patients with melioidosis have impaired function which may contribute to melioidosis-associated immune dysregulation.

Our study has several strengths. We utilized a unique whole blood assay that can be easily employed in resource-constrained settings and allows for cell stimulation prior to cryopreservation. This technique avoids some potential confounding issues experienced when isolating and studying PBMC's [51]. Additionally, our prospective study design allowed for follow-up sampling in survivors and all-cause mortality outcome measurement. Finally, all sample processing, including flow cytometry, occurred in Thailand, demonstrating the feasibility of this study design in a middle-income country. Our study also has several limitations. Patients were enrolled shortly after clinical samples were determined to be culture-positive for *B. pseudomallei* and so our findings may not be reflective of earlier time points during infection. Also, unlike many prior reports in melioidosis, we also did not specifically assess the effect of diabetes on cellular responses, a major risk factor for melioidosis acquisition but not mortality. The premade whole blood plate protocol did not allow for additional stimuli targeting specific cell populations and sufficient samples were not available for additional cell-specific markers. Finally, as noted previously, our assessment of cryopreserved whole blood, despite stimulation prior to cryopreservation, may not completely reflect cellular function in vivo.

In conclusion, patients hospitalized with melioidosis have evidence of dysregulation in both the innate and adaptive immune responses. Specifically, melioidosis survivors acutely upregulate their Th17 and effector CD8^+^ T cell functional responses and develop unique cytokine polyfunctionality. Patients with melioidosis also display an acquired impairment of monocyte function. Many of these immune response alterations resolve during recovery but some T cell functional changes persist. Further investigation is necessary to elucidate the specific mechanisms underlying melioidosis-induced immune dysregulation but could uncover novel therapeutic targets of this frequently lethal disease.

## Author contributions

SWW, PE, TEW and NC contributed to the conceptual design of the experiments. SWW, SS and PE performed the experiments. NC, RP, AD, NS, ET, KP and SK assisted in acquiring data and contributing to the analysis plan. SWW and PE analysed the data. SWW and PE wrote the initial draft of the manuscript and all authors were involved in editing and writing the final manuscript.

## Supplementary Material

melioid_cell_supp_EMI.pdf
