# Supplementary material for: Dysfunctional host cellular immune responses are associated with mortality in melioidosis
Source: Emerg Microbes Infect. 2024 Jul 15;13(1):2380822. doi: 10.1080/22221751.2024.2380822 (PMC11293272; doi:10.1080/22221751.2024.2380822)
Supplement: melioid_cell_supp_EMI.pdf [file TEMI_A_2380822_SM3542.pdf]

Dysfunctional host cellular immune responses are associated with mortality in melioidosis.

Supplementary material

Supplementary Table 1: Fluorophore-conjugated antibodies used in flow cytometry analysis. (2)
Supplementary Figure 1: Gating strategy of lymphocytes for flow cytometry. (3)
Supplementary Figure 2: Gating strategy of non-lymphocytes for flow cytometry. (4)
Supplementary Figure 3: T cell concentrations in acute melioidosis. (5-6)
Supplementary Figure 4: Innate immune cell concentrations in acute melioidosis. (7)
Supplementary Figure 5. Monocyte functional recovery after melioidosis. (8)
Supplementary Figure 6: Correlations of monocytes and lymphocytes in melioidosis. (9)

Supplementary Table 1: Fluorophore-conjugated antibodies used in flow cytometry analysis.

Target	Fluorophore	Clone	Manufacturer
CD3	Alexa 700	UCHT1	BD biosciences
CD4	PE-CD594	SK3	BD biosciences
CD8	eFluor450	SK1	eBioscience
CCR7	APC	G043H7	Biolegend
CD45RA	FITC	HI100	ebioscience
CCR6	BV650	G034E3	Biolegend
IL-17A	BV510	BL168	Biolegend
IFN-γ	PerCP	4S.B3	eBioscience
CD3	PE-CF594	UCHT1	BD biosciences
CD14	HorizonV500	MφP9	BD biosciences
HLA-DR	BV605	G46-6	BD biosciences
CD56	BV650	NCAM16.2	BD biosciences
CD16	FITC	3G8	BD biosciences
CD11c	APC	SHCL-3	BD biosciences
CD123	PE-Cy7	7G3	BD biosciences
IFN-γ	PE	4S.B3	BD biosciences
TNF-α	AlexaFluor700	MAb11	BD biosciences
IL-6	PerCP-eF710	MQ2-13A5	eBioscience
IL-12	eFluor450	C8.6	eBioscience
Live/dead	eFluor780		eBioscience

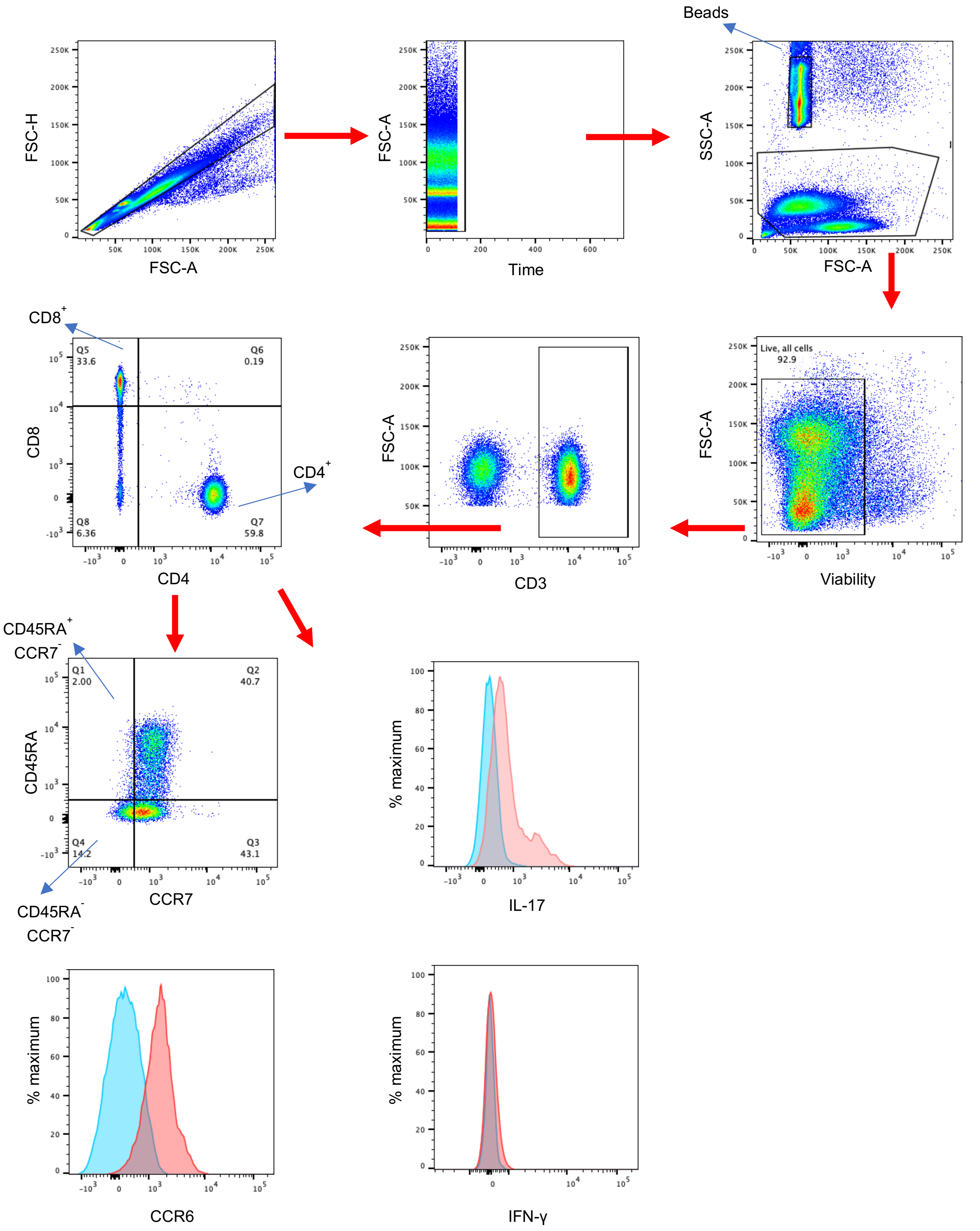

Supplementary Figure 1. Gating strategy of lymphocytes for flow cytometry. Cell surface markers were used to identify live, $CD3^{+}$ cells as lymphocytes, including CD4 and CD8+ cells. Cell surface expression of CD45RA, CCR7 as well as CCR6 were used to identify phenotypes and subsets. Cells with intracellular cytokine production of IL-17 and IFN-γ were also identified. For histograms, blue curves represent the FMO control and red curves display a representative target population.

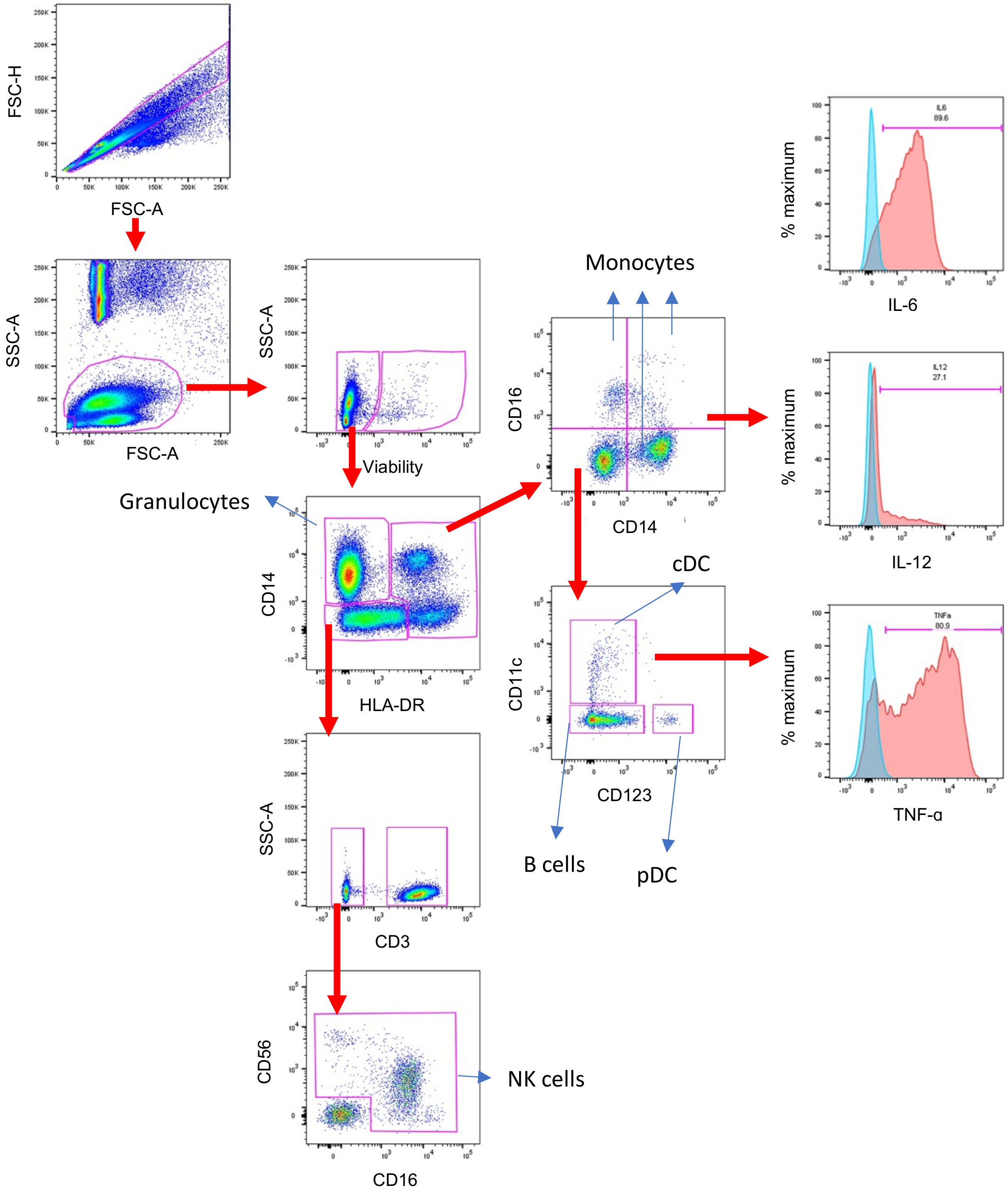

Supplementary Figure 2. Gating strategy of non-lymphocytes for flow cytometry. Cell surface markers were used to identify different types and subsets of each immune cells including granulocytes, NK cells, monocytes, conventional (cDC) and plasmacytoid (pDC) dendritic cells, and B cells. Monocytes and cDC's with intracellular cytokine production of TNF-α, IL-6 and IL-12 were also identified. For histograms, red curves represent target populations after stimulation.

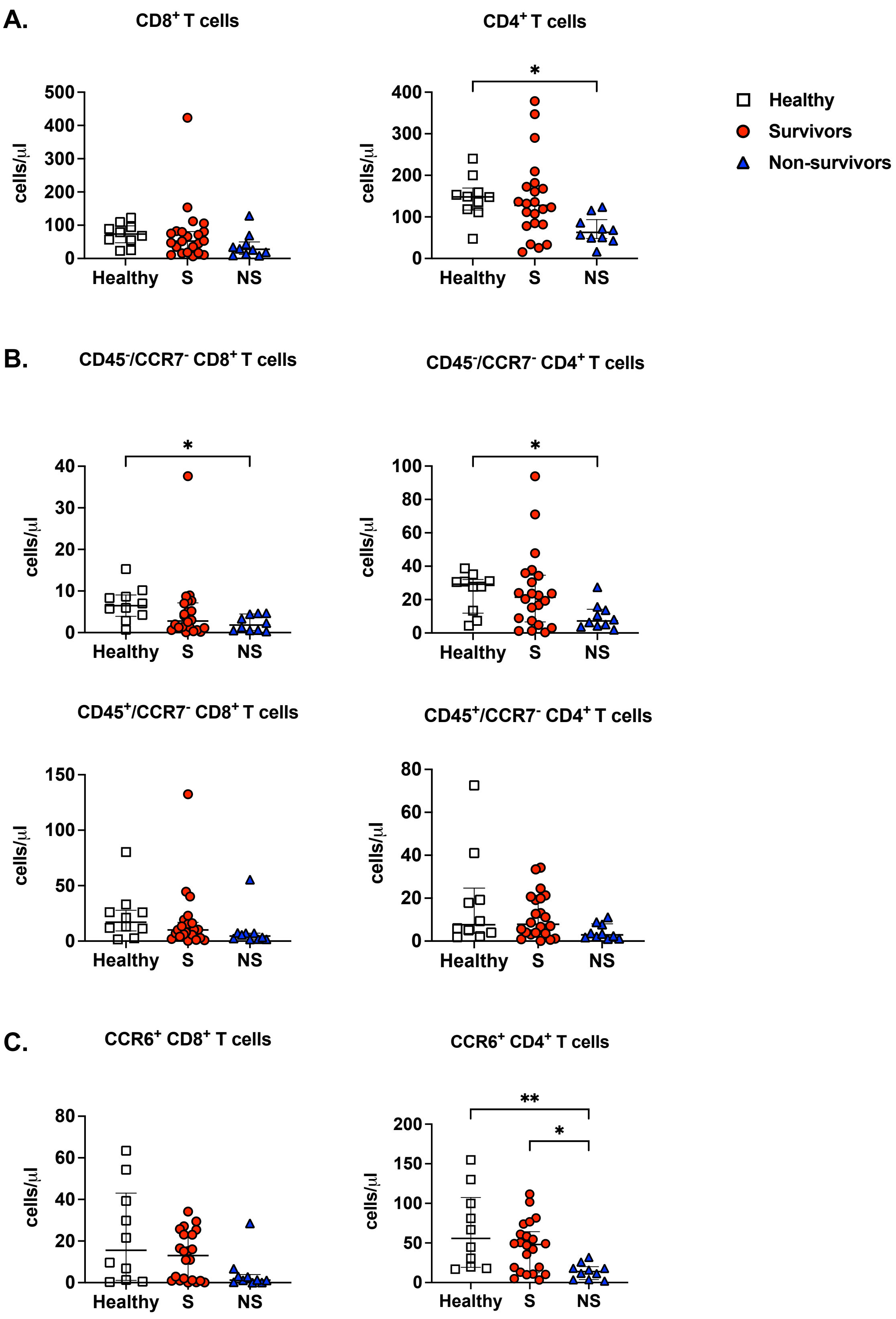
A.
CD8⁺ T cells
CD4⁺ T cells
B.
CD45⁻/CCR7⁻ CD8⁺ T cells
CD45⁻/CCR7⁻ CD4⁺ T cells
CD45⁺/CCR7⁻ CD8⁺ T cells
CD45⁺/CCR7⁻ CD4⁺ T cells
C.
CCR6⁺ CD8⁺ T cells
CCR6⁺ CD4⁺ T cells
cells/µl
Healthy
S
NS
*
**
Healthy
Survivors
Non-survivors

Supplementary Figure 3. T cell concentrations in acute melioidosis. Whole blood was obtained from patients with melioidosis within 24 hours of culture positivity (Survivors: S, N=22; 28-day non-survivors: NS, N=10) and healthy donors (Healthy, N=10). (A) Unstimulated whole blood was assessed by flow cytometry and the concentrations of different lymphocyte populations was calculated using counting beads (cells/µl). (B) The concentration of effector memory T cell phenotypes was assessed including $CD45RA^-$, $CCR7^-$ (effector memory) and $CD45RA^+$, $CCR7^-$ (terminally-differentiated effector memory). (C) T cell populations with cell surface expression of $CCR6^+$ T cells were also assessed. Median and interquartile range presented; the Kruskal-Wallis test was performed for statistical comparisons, followed by the Dunn's test for multiple comparisons. *P<0.05, **P<0.01.

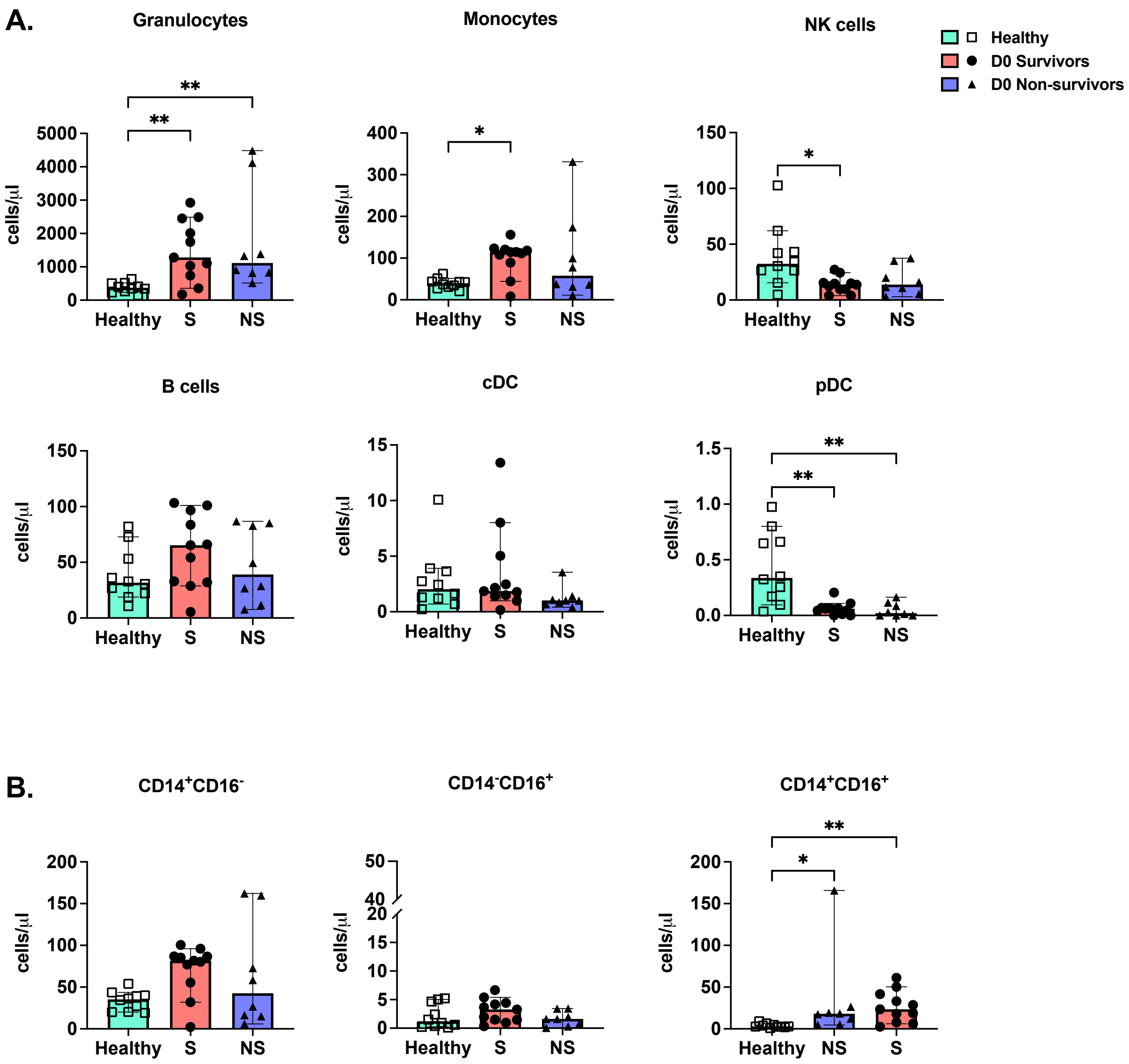

Supplementary Figure 4. Innate immune cell concentrations in acute melioidosis. Whole blood was obtained from patients with melioidosis within 24 hours of culture positivity (Survivors: S (red/circles), N=11; 28-day non-survivors: NS (purple/triangles), N=8) and healthy donors (Healthy (green/squares), N=10). (A) Unstimulated whole blood was assessed by flow cytometry and the concentrations of different immune cell populations was calculated using counting beads (cells/µl). (B) Monocyte populations including classical ($CD14^+$, $CD16^-$), non-classical ($CD14^-$, $CD16^+$) and intermediate ($CD14^+$, $CD16^+$) monocyte populations were also identified. Median and interquartile range presented; the Kruskal-Wallis test was performed for statistical comparisons of unpaired data, followed by the Dunn's test for multiple comparisons. *P<0.05, **P<0.01.

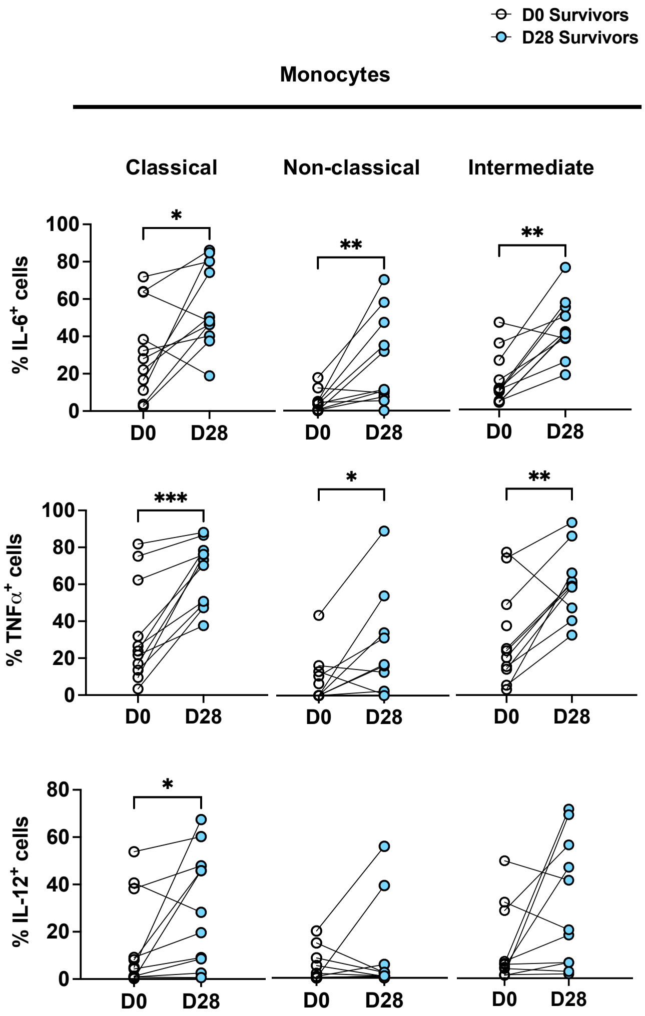

Supplementary Figure 5. Monocyte functional recovery after melioidosis. Whole blood was obtained from patients with melioidosis at enrollment (D0/open circles, N=11) and 28-days after enrollment (D28/light blue circles). Samples were immediately stimulated with Bp-LPS, cryopreserved and then stained for surface and intracellular markers and assessed by flow cytometry. Data represent the relative frequency of cytokine-producing cells for each monocyte population. The Wilcoxon test was used for comparisons of paired samples. *P<0.05, **P<0.01, ***P<0.001.

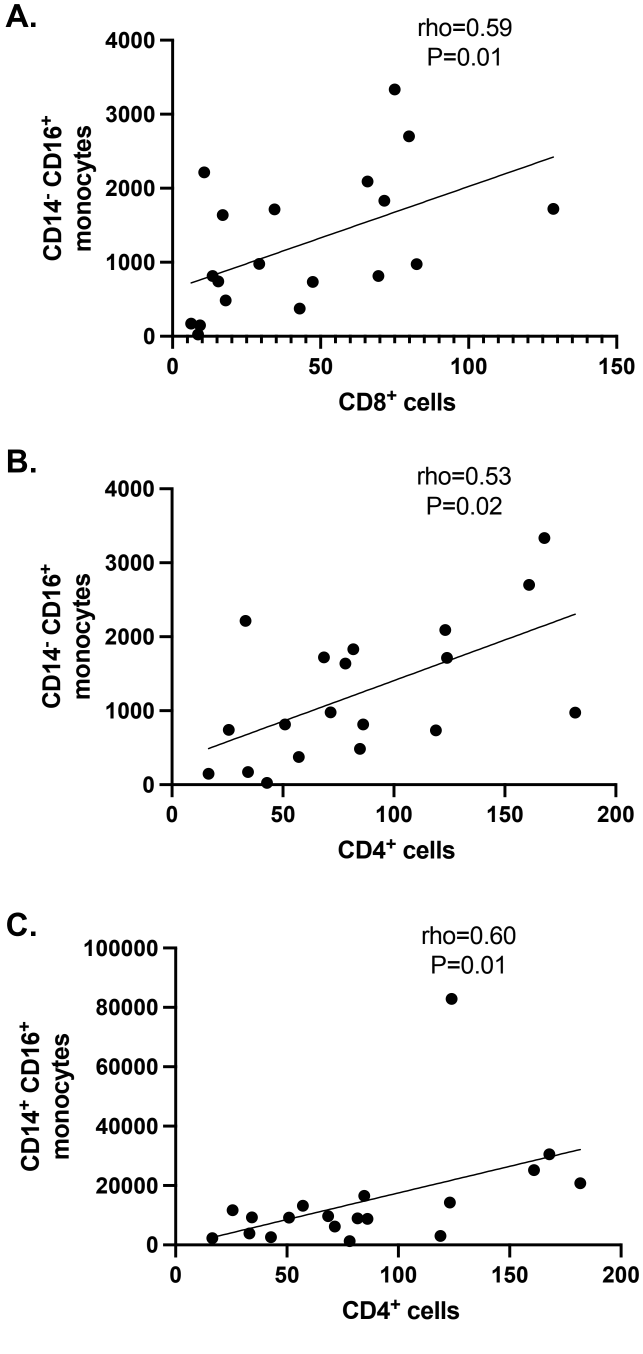

Supplementary Figure 6. Correlations of monocytes and lymphocytes in melioidosis. Whole blood was obtained from patients with melioidosis within 24 hours of culture positivity (N=19). Cells were identified by flow cytometry and cell concentrations calculated. Correlations of (A) $CD8^+$ T cells and non-classical ($CD14^-$, $CD16^+$) monocytes, (B) $CD4^+$ T cells and non-classical ($CD14^-$, $CD16^+$) monocytes, and (C) $CD4^+$ T cells and intermediate ($CD14^+$, $CD16^+$) monocytes are listed. Spearman's rho and P value presented.
